# Supplementary material for: Skin Barrier Function in Psoriasis and Atopic Dermatitis: Transepidermal Water Loss and Temperature as Useful Tools to Assess Disease Severity
Source: J Clin Med. 2021 Jan 19;10(2):359. doi: 10.3390/jcm10020359 (PMC7833436; doi:10.3390/jcm10020359)
Supplement: Supplementary file 1 [file jcm-10-00359-s001.pdf]

Journal of
Clinical Medicine

MDPI

Supplementary Material

Table S1. Homeostasis parameters between psoriatic patients and healthy participants.

Skin homeostasis parameters	Healthy skin (n = 92)	Uninvolved psoriatic skin (n = 92)	Psoriatic plaque (n = 92)	p*	p**	p***
TEWL ($g{\cdot}m^{-2}h^{-1}$)	12.34 (7.77)	12.06 (7.95)	18.45 (10.28)	0.811	<0.001**	<0.001***
SCH (AU)	44.39 (18.91)	38.43 (13.66)	8.71 (8.90)	0.015*	<0.001**	<0.001***
Temperature (ºC)	31.18 (1.05)	30.57 (1.73)	30.95 (1.55)	0.004*	0.244	0.046***
Erythema (AU)	285.91 (55.23)	311.56 (70.99)	408.44 (70.52)	0.029*	<0.001**	<0.001***
Melanin (AU)	180.19 (46.24)	238.54 (78.57)	191.10 (8.92)	<0.001*	0.312	<0.001***
pH	5.97 (0.74)	6.05 (0.62)	6.01 (0.96)	0.457	0.791	0.687
Elasticity (%)	0.75 (0.11)	0.73 (0.14)	0.75 (0.19)	0.205	0.953	0.246

AU, arbitrary units; SCH, Stratum Corneum Hydration; TEWL, Transepidermal Water Loss. * p value after using Student's t test for independent samples to compare homeostasis parameters between healthy skin and uninvolved psoriatic skin. ** p value after using Student's t test for independent samples to compare homeostasis parameters between healthy skin and psoriatic plaque. *** p value after using Student's t test for paired samples to compare homeostasis parameters between uninvolved psoriatic skin and psoriatic plaque.

Table S2. Homeostasis parameters between atopic dermatitis patients and healthy participants.

Skin homeostasis parameters	Healthy skin (n = 65)	Uninvolved AD skin (n = 65)	AD eczematous lesion (n = 65)	p*	p**	p***
TEWL ($g{\cdot}m^{-2}h^{-1}$)	11.60 (7.78)	13.15 (7.92)	28.68 (14.28)	0.296	<0.001**	<0.001***
SCH (AU)	50.73 (21.78)	40.95 (16.03)	25.20 (18.28)	0.007*	<0.001**	<0.001***
Temperature (ºC)	31.37 (1.71)	31.35 (1.27)	32.05 (1.30)	0.956	0.017**	<0.001***
Erythema (AU)	244.44 (43.92)	244.50 (74.05)	387.21 (83.06)	0.997	<0.001**	<0.001***
Melanin (AU)	165.30 (30.83)	204.10 (77.42)	210.37 (98.37)	0.017*	0.023**	0.557
pH	5.75 (0.72)	5.91 (0.54)	5.94 (0.53)	0.207	0.112	0.442
Elasticity (%)	0.76 (0.10)	0.74 (0.15)	0.69 (0.17)	0.319	0.023**	0.038***

AD, atopic dermatitis; AU, arbitrary units; SCH, Stratum Corneum Hydration; TEWL, Transepidermal Water Loss. *p value after using Student's t test for independent samples to compare homeostasis parameters between healthy skin and uninvolved AD skin. ** p value after using Student's t test for independent samples to compare homeostasis parameters between healthy skin and AD eczematous lesion. *** p value after using Student's t test for paired samples to compare homeostasis parameters between uninvolved AD skin and AD eczematous lesion.
